# Determination of the best method to estimate glomerular filtration rate from serum creatinine in adult patients with sickle cell disease: a prospective observational cohort study

**DOI:** 10.1186/1471-2369-13-83

**Published:** 2012-08-06

**Authors:** Jean-Benoît Arlet, Jean-Antoine Ribeil, Gilles Chatellier, Dominique Eladari, Sophie De Seigneux, Jean-Claude Souberbielle, Gérard Friedlander, Marianne de Montalembert, Jacques Pouchot, Dominique Prié, Marie Courbebaisse

**Affiliations:** 1 Service de Médecine Interne, centre de référence des syndromes drépanocytaires majeurs, Faculté de médecine Paris Descartes et Assistance publique – Hôpitaux de Paris, Hôpital Européen Georges Pompidou, Cedex 15, Paris, 75908, France; 2 Département de Biothérapie, centre de référence des syndromes drépanocytaires majeurs, Faculté de médecine Paris Descartes et Assistance publique – Hôpitaux de Paris, Hôpital Necker Enfants Malades, 161, rue de Sèvres, 75015 Paris Cedex 15, Paris, France, 75908, France; 3 Faculté de médecine Paris Descartes et CIC-EC4 INSERM, Assistance publique – Hôpitaux de Paris, Hôpital Européen Georges Pompidou, Cedex 15, Paris, 75908, France; 4 Service d’Explorations Fonctionnelles. Faculté de médecine Paris Descartes, INSERM U872 et Assistance publique –Hôpitaux de Paris, Hôpital Européen Georges Pompidou, 20, rue Leblanc, Cedex 15, Paris, 75908, France; 5 Service de Néphrologie, Hôpital Cantonal de Genève, 4 rue Perret Gentil, Genève 4, Suisse, 1211, Switzerland; 6 Service d’Explorations Fonctionnelles. Faculté de médecine Paris Descartes, INSERM U845 et Assistance publique –Hôpitaux de Paris, Hôpital Necker Enfants Malades, 161, rue de Sèvres, 75015 Paris, Cedex 15, France, Paris, 75908, France; 7 Service d’Explorations Fonctionnelles. Faculté de médecine Paris Descartes, INSERM U845 et Assistance publique –Hôpitaux de Paris, Hôpital Européen Georges Pompidou, 20, rue Leblanc Cedex 15, Paris, 75908, France; 8 Service de Pédiatrie Générale, Assistance publique –Hôpitaux de Paris, Hôpital Necker Enfants Malades, 161, rue de Sèvres, 75015 Paris, Cedex 15, France, Paris, 75908, France; 9 Hôpital Européen Georges Pompidou, 20, rue Leblanc, Cedex 15, Paris, 75908, France

**Keywords:** Sickle cell disease, Glomerular hyperfiltration, Albuminuria, Glomerular filtration rate, CKD-EPI equation, Iohexol plasma clearance, Ethnicity

## Abstract

**Background:**

Sickle cell disease (SCD) leads to tissue hypoxia resulting in chronic organ dysfunction including SCD associated nephropathy. The goal of our study was to determine the best equation to estimate glomerular filtration rate (GFR) in SCD adult patients.

**Methods:**

We conducted a prospective observational cohort study. Since 2007, all adult SCD patients in steady state, followed in two medical departments, have had their GFR measured using iohexol plasma clearance (gold standard). The Cockcroft-Gault, MDRD-v4, CKP-EPI and finally, MDRD and CKD-EPI equations without adjustment for ethnicity were tested to estimate GFR from serum creatinine. Estimated GFRs were compared to measured GFRs according to the graphical Bland and Altman method.

**Results:**

Sixty-four SCD patients (16 men, median age 27.5 years [range 18.0-67.5], 41 with SS-genotype were studied. They were Sub-Saharan Africa and French West Indies natives and predominantly lean (median body mass index: 22 kg/m^2^ [16-33]). Hyperfiltration (defined as measured GFR >110 mL/min/1.73 m^2^) was detected in 53.1% of patients. Urinary albumin/creatinine ratio was higher in patients with hyperfiltration than in patients with normal GFR (4.05 mg/mmol [0.14-60] *versus* 0.4 mg/mmol [0.7-81], p = 0.01). The CKD-EPI equation without adjustment for ethnicity had both the lowest bias and the greatest precision. Differences between estimated GFRs using the CKP-EPI equation and measured GFRs decreased with increasing GFR values, whereas it increased with the Cockcroft-Gault and MDRD-v4 equations.

**Conclusions:**

We confirm that SCD patients have a high rate of glomerular hyperfiltration, which is frequently associated with microalbuminuria or macroalbuminuria. In non-Afro-American SCD patients, the best method for estimating GFR from serum creatinine is the CKD-EPI equation without adjustment for ethnicity. This equation is particularly accurate to estimate high GFR values, including glomerular hyperfiltration, and thus should be recommended to screen SCD adult patients at high risk for SCD nephropathy.

## Background

Sickle cell disease (SCD) is one of the most common genetic hemoglobinopathies in which sickle hemoglobin leads to tissue hypoxia causing acute tissue damage and chronic organ dysfunction including SCD associated nephropathy [1]. Four genotypes—sickle cell anemia (HbSS), sickle-hemoglobin C disease (HbSC), and two types of sickle-β-thalassemia (Sβ + −thalassemia and Sβo-thalassemia)—account for most cases of SCD. Compared to patients with other genotypes, those with a homozygous SS genotype have more profound anemia and higher morbidity and mortality [1,2]. SCD mainly affects natives of Sub-Saharan Africa, the West Indies, India, and South-America. Because of past and more recent migratory movements and thanks to better care in childhood, SCD has become a real health issue in European countries and especially in France where more than 7000 subjects are affected, half of whom are adults [2]. Glomerular hyperfiltration seems to be one of the first steps of SCD associated nephropathy, as in type I diabetes mellitus associated nephropathy [3], and is a frequent feature in young adult SCD patients [4,5]. Considering the negative impact of SCD associated nephropathy on the prognosis and the potential interest of an early nephroprotective treatment with angiotensin converting enzyme (ACE) inhibitors [1,6,7], an accurate screening of glomerular hyperfiltration is essential. CKD-EPI, a new equation to estimate glomerular filtration rate (GFR) from serum creatinine, has been reported to be particularly accurate to estimate high levels of GFR [8] but has never been evaluated in SCD patients.

The main objective of our study was to determine the best equation to estimate GFR in SCD adult patients using five different equations. We also aimed at determining the prevalence of hyperfiltration and albuminuria among these patients and the relationship between albuminuria and GFR.

## Methods

### Patients

We conducted a prospective observational cohort study. Since January 2007, all newly referred SCD adult patients seen in two medical departments have had a comprehensive work-up including GFR measurement. At the time of investigation, all patients had to have been in steady state for at least three months (no acute illness, no vaso-occlusive crisis, no acute chest syndrome, and no urinary tract infection). Pregnant and breast feeding women, patients allergic to iodine, patients with diabetes mellitus, hypertension or other diseases susceptible to induce chronic kidney disease were not eligible for the present study. The study protocol conforms to the ethical guidelines of the 1975 Declaration of Helsinki and was approved by a local ethic committee (Comité de Protection des Personnes, Ile de France II) and received the number **2011531-RCEB**.

### Glomerular filtration rate measurement

All patients underwent direct measurement of GFR using plasma clearance of iohexol, an exogenous marker, as previously described [9]. All patients received a 5 mL intravenous dose of iohexol. Each patient then simultaneously ingested 150 mL of water within 30 minutes. Blood samples were taken at 0, 60, 120, 180, 240, and 300 minutes after injection. Clearance of iohexol was calculated by the following formula: Clearance = Dose/AUC, where AUC is the area under the plasma concentration curve.

Measured GFR (mGFR) was normalized in mL/minute/1.73 m^2^ by using the Dubois formula for the calculation of BSA (body surface area) [10].

(1) $$\text{BSA}\left({\text{m}}^{2}\right)\;=\;0,0071184\times {\text{height}}^{0,725}\times {\text{weight}}^{0,425}$$

Since there is no consensus to define glomerular hyperfiltration we chose to define glomerular hyperfiltration as mGFR higher than 110 mL/min/1.73 m^2^, as did Haymann et al. [5].

### Biological measurements

Other biological measurements included hemoglobin and reticulocyte counts, serum creatinine, and urinary albumin excretion rate (AER) on a single urinary spot expressed as mg/mmol urinary creatinine. AER was categorized as normoalbuminuria (AER < 3 mg/mmol), microalbuminuria (AER from 3 to 30 mg/mmol), or macroalbuminuria (AER > 30 mg/mmol). All measurements were made using standard hospital laboratory methods. Serum and urine creatinine were measured by using an alkaline picrate rate-blanked compensated kinetic assay (Hitachi 917 analyzer; Roche Diagnostics) with standardization to isotope dilution mass spectrometry.

### Equations used to estimate GFR

#### MDRD-v4

**(**Four variables Modification of Diet in Renal Disease equation) [11].

(2) $$\text{GFR}\;\left(\text{mL}/\text{min}/1.{73\;\text{m}}^{2}\right)=175\times \left\{{\left[\text{plasma creatinine}\;\left(\mu \text{mol}/1\right)/88.4\right]}^{-1.154}\right\}\times \text{age}\;{\left(\text{years}\right)}^{-0.203}\times 0.742\;\left(\text{if female}\right)\times 1.212\;\left(\text{if black}\right)$$

#### MDRD without adjustment for ethnicity

(3) $$\text{GFR}\;\left(\text{mL}/\text{min}/1.{73\;\text{m}}^{2}\right)=175\times \left\{{\left[\text{plasma creatinine}\;\left(\mu \text{mol}/\text{l}\right)\;/\;88.4\right]}^{-1.154}\right\}\times \text{age}\;{\left(\text{years}\right)}^{-0.203}\times 0.742\;\left(\text{if female}\right)$$

#### CKD-EPI [12]

The CKD-EPI (Chronic Kidney Disease Epidemiology Collaboration) equation, expressed as a single equation, is: ‘$\text{GFR}\;\left(\text{mL}/\text{min}/1.73\;{\text{m}}^{2}\right)=141\times \text{min}{\left(\text{Scr}/k,\;1\right)}^{\alpha }\times \text{max}{\left(\text{Scr}/k,\;1\right)}^{-1.209}\times 0.993^{\text{Age}}\times 1.018\;\left(\text{if female}\right)\times 1.159\;\left(\text{if black}\right)$, where Scr is serum creatinine, *k* is 0.7 for females and 0.9 for males, α is −0.329 for females and −0.411 for males, min indicates the minimum of Scr/*k* or 1, and max indicates the maximum of Scr/*k* or 1.’

#### CKD-EPI without adjustment for ethnicity

(4) $$\text{GFR}\;\left(\text{mL}/\text{min}/1.73\;{\text{m}}^{2}\right)=141\times \text{min}{\left(\text{Scr}/k,\;1\right)}^{\alpha }\times \text{max}{\left(\text{Scr}/k,\;1\right)}^{-1.209}\times 0.993^{\text{Age}}\times 1.018\;\left(\text{if female}\right)$$

The MDRD-v4- and CKD-EPI-derived eGFRs with or without adjustment for ethnicity are expressed as mL/min/1.73 m^2^ because the equations were derived by comparison with iothalamate-measured GFR, which itself is expressed as mL/min/1.73 m^2^.

#### Modified Cockcroft–Gault [13]

(5) $$\text{GFR}\;\left(\text{mL}/\text{min}\right)\;\text{for males}=\left[\text{gX}\left(140-\text{age}\right)\text{X weight}\left(\text{kg}\right)\right]/\text{plasma creatinine}\left(\mu \text{mol}/\text{l}\right)\text{,}$$

Where g = 1.23 for males and 1.04 for females.

Estimated GFR derived by using the Cockcroft–Gault equation was converted from mL/min to mL/min/1.73 m^2^ by multiplying calculated values by 1.73, and dividing by BSA.

### Statistical methods

Agreement between GFR estimated using the different equations described above and GFR measurement by iohexol plasma clearance (reference method) was assessed graphically by plotting the difference in GFR (estimated GFR - GFR measured by the reference method) against the mean GFR, where mean GFR is (GFR measured by the reference method + estimated GFR)/2), according to the method described by Bland and Altman [14]. Bias was estimated by the mean difference in GFR and limits of agreement defined by the mean difference ± 1.96 standard deviations of the difference (SD).

Percentages were compared using the CHI-squared test, or the Fisher test, as appropriate.

Distributions were estimated using a kernel density distribution. Bandwith selection was done using the Sheather-Jones method [15]. Calculations were made using the the KDE procedure of the SAS statistical software.

Several means were compared using the Kruskall-Wallis method (more than 2 groups) or the Mann–Whitney test (two groups). Differences were assessed using the paired t test. A p value less than 0.05 was considered significant.

In order to assess relationships between relevant quantitative variables, we used the Pearson correlation coefficient. Calculations were performed using the Statview Statistical and the SAS statistical software version 9.2.

## Results

### Description of the population and of SCD associated nephropathy

From January 2007 to December 2008, 67 consecutive adult SCD patients were studied. Three patients were excluded, including one with diabetes mellitus and two with hypertension. Finally, 64 patients were included in the present study: 41 with SS genotype, 15 with SC genotype, 7 with Sβ genotype, and 1 with SD genotype. Table 1 summarizes the main clinical and biological characteristics of the patients. They were predominantly young and lean (median body mass index (BMI): 22 kg/m^2^, range [16-33]). Most of them were native either of Sub-Saharan Africa or of the French West Indies.

**Table 1:** Characteristics of the studied population

	**Whole population (n = 64)**	**Patients with SS genotype (n = 41)**	**Patients with non- SS genotype (n = 23)**	p value * SS vs non-SS
	(n = 64)	(n = 41)	(n = 23)	
**Sex ratio** (M/F), (M%)	16/48 (25.0%)	11/30 (27%)	5/18 (22%)	0.22
**Age**, years	27.5 [18–67.5]	26.8 [18–49.5]	31.7 [18–67.5]	0.03
**Ethnical origin**				0.74
*Sub Saharian Africa*	48 (75%)	32 (78.1%)	16 (69.6%)	
*French West Indies*	10 (15.6%)	6 (14.6%)	4 (17.4%)	
*Maghreb*	2 (3.1%)	1 (2.4%)	1 (4.3%)	
*Other*	4 (6.3%)	2 (4.9%)	2 (8.7%)	
**Height**, m	1.67 [1.48-1.83]	1.68 [1.53-1.83]	1.66 [1.48-1.78]	0.27
**Weight**, kg	63 [43.5-90]	61.5 [43.5-81]	64 [45–90]	0.03
**Body mass index**, kg/m^2^	22 [16–33]	21 [16–30]	24 [16–33)]	0.017
**Hemoglobin**, g/dL	8.9 [5.5-14.3]	8.2 [5.5-12.6]	10.7 [7.5-14.3]	<0.001
**Reticulocyte count**, ×10^3^/mm3	170 [32–466]	219 [32–466]	99 [34–271]	<0.001
**Plasma creatinine**, μmol/L	55 [27–113]	48 [27–76]	65 [51–113]	<0.001
**mGFR**, mL/min/1,73 m^2^	112.5 [29–183]	119 [65–183]	98 [29–163]	<0.002
*GFR >110 mL/min/1,73 m*^*2*^	34 (53.1%)	28 (68.3%)	6 (26.1%)	0,0017
**Urinary albumin/creatinine**, mg/mmol	2.87 [0.07-134]	4.84 [0.14-80.6]	0.63 [0.07-134]	0.005
*No albuminuria (<3 mg/mmol)*	32 (50%)	16 (39%)	16 (69.5%)	
*Microalbuminuria*	23 (36%)	17 (41.5%)	6 (26.1%)	
*Macroalbuminuria (>30 mg/mmol)*	9 (14%)	8 (19.5%)	1 (4.3%)	0 .053

Results are expressed as numbers (%) or median [range].

M: male, F: female,

mGFR: glomerular filtration rate measured by iohexol plasma clearance.

* Tests are either Chi-square or Fisher’s exact test for categorical variables or Kruskall-Wallis test (more than two groups) or the Mann–Whitney test (two groups) for continuous variables.

Thirty-four patients (53.1%) had hyperfiltration (defined as measured GFR (mGFR) >110 mL/min/1.73 m^2^). Measured GFR non-indexed for BSA was comparable to mGFR expressed per BSA (mGFR non-indexed BSA = 110.3 mL/min (median, 26.8-167.9); mGFR expressed per BSA = 112.5 mL/min/1.73 m^2^ (29–183); p = 0.2). Hyperfiltration was more common in patients with SS genotype than among those with non-SS genotype (p = 0.0017) (Table 1). Only one patient (a 48-year-old female with SC-genotype) had a GFR <60 mL/min/1.73 m^2^. Microalbuminuria or macroalbuminuria were found in 36% and 14% of the patients, respectively. As shown in Table 1, the median urinary albumin/creatinine ratio was significantly higher in patients with hyperfiltration than in patients with normal or low mGFR (4.05 mg/mmol [0.14-60] *versus* 0.4 mg/mmol [0.7-81], p = 0.01). As shown in Table 2, when measured GFR is divided into quartiles, the median urinary albumin/creatinine ratio was the lowest for the second quartile of mGFR and significantly increased for mGFR above 112 mL/min/1.73 m^2^ (p = 0.029).

**Table 2:** Urinary albumin/creatinine ratio according to measured glomerular filtration rate (mGFR) divided into quartiles

**mGFR**	**Quartile range (mL/min/1.73 m**^**2**^**)**	**Median within the quartile (mL/min/1.73 m**^**2**^**)**	**Urinary albumin/creatinine ratio, (mg/mmol)**	p value*
Quartile 1	[29–75]	74.5	1.86 [0.23-134.24]	0.029
Quartile 2	[96–112]	105	0.405 [0.07-80.63]	
Quartile 3	[113–128]	119	3.04 [0.14-31.47]	
Quartile 4	[129–183]	145	8.43 [0.36-59.77]	

mGFR: glomerular filtration rate measured by iohexol plasma clearance.

Q1, Q2, Q3, Q4: each quartile of mGFR.

* p value was calculated using a Kruskal-Wallis test. Results are expressed as median [range].

### Determination of the best equation to estimate GFR from plasma creatinine in adult patients with SCD

**Figure 1:**
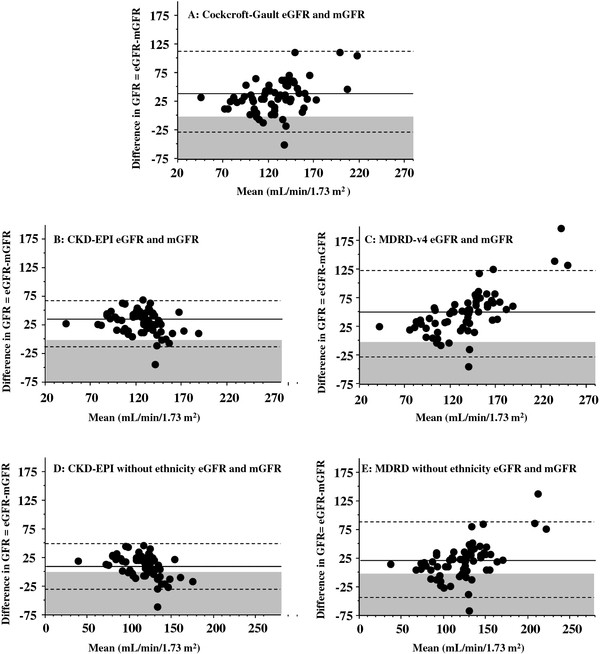
**Bland and Altman plots for glomerular filtration rate (GFR) estimated with different equations compared to measured GFR.** Each graph is a Bland and Altman plot comparing a specific equation used for GFR estimation to the reference method (GFR measured using iohexol plasma clearance). Mean GFR is calculated as follows: Mean GFR = (measured GFR + estimated GFR)/2. The plain line represents the mean difference between estimated GFR (eGFR) and GFR measured using the reference method (mGFR). In the grey zone, eGFR is lower than mGFR. Dashed lines represent ± 1.96 SD. Results are expressed as mL/min/1.73 m^2^.

**Figure 2:**
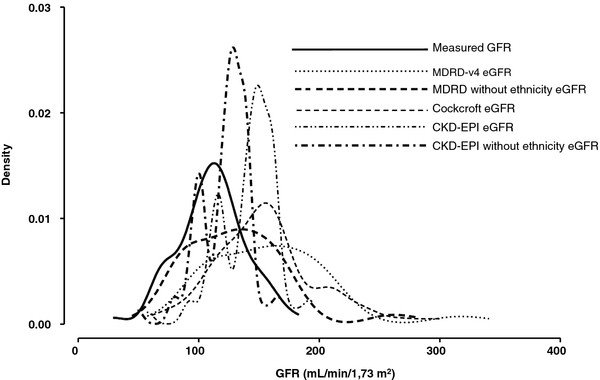
**Distributions of measured GFR and estimated GFRs using a kernel density distribution.** CKD: Chronic Kidney Disease; CKD-EPI: Chronic Kidney Disease Epidemiology Collaboration; eGFR: estimated GFR; GFR: Glomerular Filtration rate; MDRD: Modification of Diet in Renal Disease. Results of GFR are expressed as mL/min/1.73 m^2^.

Bland and Altman graphs are presented in Figure 1. In our adult SCD population, all equations overestimate GFR compared to mGFR by iohexol plasma clearance (p < 0.05, paired t-test). Moreover, the wide limits of agreement (Table 3) suggest that large discrepancies between equations and mGFR can be observed. Distributions of measured GFR and estimated GFRs using a kernel density distribution were represented in Figure 2 and confirm this previous point. Compared to the Cockcroft and Gault and MDRD-v4 equations, the CKD-EPI equation had both the lowest bias and the narrowest limits of agreement. The difference between estimated GFR calculated with the CKD-EPI equation and mGFR decreases with increasing GFR values (r = − 0.23, p = 0.06). In addition, we observed a significant relationship between the difference and the mean for both the Cockcroft and Gault (r = 0.34, p < 0.05) and the MDRD-v4 equations (r = 0.68, p < 0.001). This means that the difference between estimated GFR and mGFR (gold standard) increases with increasing GFR values.

**Table 3:** Mean difference (95% CI) and median difference [IQR] between each method of glomerular filtration rate (GFR) estimation and measured GFR

**Method of estimation**	**Mean difference**	**95% Confidence interval**	p value*
	***Median***	***[IQR]***	
Cockcroft–Gault	**45.3**	**26.8 to 41.3**	**<0.0001**
	*38.9*	*[20.5-63.8]*	
CKD-EPI	**30.2**	**25.8 t o 35.2**	**<0.0001**
	*30.5*	*[16.5-44.3]*	
CKD-EPI without adjustment for ethnicity	**10.7**	**5.8 to 15.7**	**<0.0001**
	*12.8*	*[−0.7-24.8]*	
MDRD-v4	**48.7**	**40.0 to 58.4**	**<0.0001**
	*49.3*	*[24.7-64.8]*	
MDRD without adjustment for ethnicity	**20.7**	**12.9 to 28.5**	**<0.0001**
	*19.9*	*[4.9-32.9]*	

Results are expressed as mL/min/1.73 m^2^.

IQR: Inter Quartile Range.

CKD: Chronic Kidney Disease.

MDRD: Modification of Diet in Renal Disease.

CKD-EPI: Chronic Kidney Disease Epidemiology Collaboration.

* p value was calculated using a paired t-test.

The MDRD-v4 and CKD-EPI equations comprise four variables: age, sex, plasma creatinine and White/African-American ethnic group. The patients of our study population were mainly natives of Sub-Saharan African countries and of the French West Indies. None of our patients was of African-American origin. Consequently, the adjustment for racial group was not considered appropriate for our population. Therefore, we removed ethnicity from these two equations. Without this variable (Figures 1D and 1E), overestimation decreased for both MDRD-v4 and CKD-EPI whereas limits of agreement remained comparable. Among the five equations tested to estimate GFR, the CKD-EPI equation without adjustment for ethnic group had both the lowest bias and the narrowest limits of agreement. Finally, for the CKD-EPI equation without adjustment for ethnic group, the difference with the gold standard decreased with increasing GFR values (r = − 0.43, p < 0.001), whereas this difference increased for the MDRD equation without adjustment for ethnic group (r = 0.538, p < 0.001).

## Discussion

Our study shows that the CKD-EPI equation without the adjustment for African-American ethnicity is the best equation to estimate GFR from serum creatinine in adult SCD originating from Sub-Saharan Africa and the French West Indies. It also confirms the high prevalence of hyperfiltration among these patients and its association with increased urinary albumin excretion rate.

Recently, Haymann et al. have reported that the MDRD-v4 equation was a more robust predictor of hyperfiltration compared to the Cockcroft and Gault estimated GFR in a cohort of adult SCD patients, although the MDRD-v4 equation systematically overestimated measured GFR [5]. In our study, we clearly show that the MDRD-v4 equation has both the highest bias and the lowest precision, followed by the Cockcroft and Gault equation and lastly by the CKD-EPI equation, not assessed in the study by Haymann et al. [5]. In the MDRD study [11,16], the MDRD-v4 equation was found to be accurate in predicting GFR for values <60 mL/min/1.73 m^2^ whereas the CKD-EPI equation was shown to be as accurate as MDRD in the subgroup with estimated GFR <60 mL/min/1.73 m^2^ and substantially more accurate in the subgroup with estimated GFR >60 mL/min/1.73 m^2^[12,17,18]. The better performance of the CKD-EPI equation in this specific SCD population is thus expected at least in part because many such patients have normal or high GFR [12].

We also showed that both the MDRD-v4 and the CKD-EPI equations gave better estimation of GFR after excluding the correction for ethnicity. Finally, among the five equations tested, the CKD-EPI equation without adjustment for ethnicity was the most accurate to estimate GFR in our population. The correction of estimated GFR for black people by multiplying estimated GFR by 1.212 for the MDRD-v4 equation [11] and by 1.159 for the CKD-EPI equation [12] is based on studies performed in African-Americans. It has not been validated in black people of other ethnic origin, nor at extremes of body weight [8]. It has recently been shown that the CKD-EPI equation without adjustment for ethnicity is the most useful equation to estimate GFR in a lean Sub-Saharan African population [19] which may share some characteristics with adult SCD populations. Moreover, in a study of one hundred black South Africans, Van Deventer et al. have reported that both the MDRD-v4 [20] and the CKD-EPI [21] equations overestimated GFR when using the ethnicity correction factor as suggested for African-Americans. Among the patients they studied, fifteen had a BMI < 20 kg/m^2^ and their median weight and BSA were 67 kg and 1.75 m^2^ respectively. In our study, the unexpected improvement of the performance of the MDRD and CKD-EPI equations without adjustment for ethnicity to estimate GFR could be explained by the fact that our patients had lower BMI compared to the one of the patients tested in MDRD and CKD-EPI samples since the mean body weight was 79.6 kg in the MDRD study [11] and 82 kg in the CKD-EPI study [12]. Another explanation could be that meat intake [22] may be lower in our population than in the African-American one. It was also shown that African-Americans had greater serum creatinine levels and urinary creatinine excretion for any given GFR compared to non-African-Americans [23]. Finally, the MDRD and CKD-EPI equations were developed in patients with chronic kidney disease stage 4–5. The study of Peralta et al. [24] strongly suggests that even in a cohort of African-American, the race correction factors of 1.21 for the MDRD-v4 et 1.16 for the CKD-EPI equations are probably too high for young patients with CKD-EPI estimated GFR comprised between 60 and 80 mL/min/1.73 m^2^ and should rather be 1.12.

Determining the best equation for GFR estimation is of great importance, especially for the care of SCD patients living in developing areas where GFR measurement is not easily accessible. Our results, as well as those of other studies [19,21], claim for the use of more specific equations to estimate GFR according to the sub-population tested. Most online formulas for calculating estimated GFR using the MDRD-v4 or CKD-EPI equations propose to choose between “black skin” and “non black skin” or between “African origin” and “non- African origin”, whereas they should offer the choice of “African-American” *versus* “non- African-American origin”.

In the case of SCD patients, screening for GFR level and especially for glomerular hyperfiltration status is of paramount importance, given its association with microalbuminuria or macroalbuminuria [5,25,26]. As previously explained, we chose to define glomerular hyperfiltration as mGFR higher than 110 mL/min/1.73 m^2^, as did Haymann et al. [5]. Using this controversial definition, glomerular hyperfiltration seems to be a very frequent finding in young adult SCD patients: in our study, 68% of patients with SS-genotype had glomerular hyperfiltration, similar to the 66% of 48 homozygous SCD patients reported previously for whom GFR was measured using urinary ^51^Cr EDTA method [5]. Moreover, in our patients’ population, we demonstrated that the urinary albumin/creatinine ratio significantly increased when mGFR was above 112 mL/mn/1.73 m^2^. Since we showed that mGFR non-indexed for BSA was comparable to mGFR expressed per BSA, we can assert that the putative hyperfiltration status is not an artifact linked to BSA indexation in our population. Indeed BSA indexation is questionable especially in subjects with low BMI [27,28]. However, both the MDRD and CKD-EPI equations automatically estimate GFR expressed per BSA, so that we could not express estimated and measured GFRs without this indexation.

One of the limitations of our study could lie in the lack of homogeneity of our population since we chose to pool the SCD patients with SS and non-SS genotypes. However, although the patients with hemoglobin SS had a more severe disease than those with other sickling hemoglobinopathies, the measurement properties of the five equations tested were similar in SCD patients with or without the SS genotype. Ideally, the validity of the CKD-EPI equation without the adjustment for African-American ethnicity should have been assessed in a control group comprising individuals of the same ethnic origin but with an AA genotype test to allow us to claim that hyperfiltration was specific to SCD but this last point was not the main goal of our study. Moreover, Thompson et al. already have already shown that SCD patients have higher GFR as well as higher urinary albumin to creatinine ratio than controls [29].

Another limitation of our study is the definition of hyperfiltration. We chose to consider that a measured GFR higher than 110 mL/min/1.73 m^2^ defines hyperfiltration for two reasons: first, this definition is the one given by Haymann et al. in their recent work about GFR in SCD patients [5] and we wished to compare our results to theirs; secondly, although this arbitrary level may be considered as too low, we observed that in our population, urinary albumin excretion was the lowest when mGFR was between 96 and 112 mL/min/1.73 m^2^, whereas urinary albumin excretion significantly increased when mGFR was higher than112 mL/min/1.73 m^2^. Consequently a mGFR higher than 110 mL/min/1.73 m^2^ may be considered as pathological in this population as it is more frequently associated with the presence of micro or macroalbuminuria.

## Conclusions

Our study confirms that SCD patients have a high rate of glomerular hyperfiltration, which is frequently associated with microalbuminuria or macroalbuminuria and shows that in SCD patients of non African-American origin, the CKD-EPI equation without adjustment for ethnicity should be the recommended method to estimate GFR.

## Abbreviations

ACE: angiotensin converting enzyme; AER: albumin excretion rate; BMI: body mass index; BSA: body surface area; CKD: chronic kidney disease; eGFR: estimated glomerular filtration rate; mGFR: measured glomerular filtration rate; SCD: sickle cell disease CKD: Chronic Kidney Disease; MDRD: Modification of Diet in Renal Disease; CKD-EPI: Chronic Kidney Disease Epidemiology Collaboration.

## Competing interests

The authors declare that they have no competing interests.

## Authors' contributions

**J-B A** contributed to patients’ recruitment, to acquisition and interpretation of data and to the manuscript preparation. **J-A R and J P** contributed to patients’ recruitment and to the manuscript preparation. **G C** performed statistical analysis and contributed to the manuscript preparation. **D E and D P** performed GFR measurements and contributed to interpretation of data. **S D S** contributed to the manuscript preparation. **J-C S and G F** contributed to acquisition and interpretation of data. **M C** performed GFR measurement, contributed to acquisition and interpretation of data and wrote the manuscript. All authors gave final approval of the present version.

## Pre-publication history

The pre-publication history for this paper can be accessed here:

http://www.biomedcentral.com/1471-2369/13/83/prepub
